# Assessing Gear Modifications Needed to Optimize Yields in a Heavily Exploited, Multi-Species, Seagrass and Coral Reef Fishery

**DOI:** 10.1371/journal.pone.0036022

**Published:** 2012-05-04

**Authors:** Christina C. Hicks, Timothy R. McClanahan

**Affiliations:** 1 Australian Research Council Centre of Excellence for Coral Reef Studies, James Cook University, Townsville, Queensland, Australia; 2 Coral Reef Conservation Project, Mombasa, Kenya; 3 Marine Programs, Wildlife Conservation Society, New York, New York, United States of America; Swansea University, United Kingdom

## Abstract

Effective management is necessary if small-scale fisheries, such as those found in mixed habitats including seagrass and coral reefs, are to continue providing food for many of the poorest communities of the world. Gear-based management, although under represented and under studied, has the potential to be adaptive, address multiple objectives, and be crafted to the socio-economic setting. Management effectiveness in seagrass and coral reef fisheries has generally been evaluated at the scale of the fish community. However, community level indicators can mask species-specific declines that provide significant portions of the fisheries yields and income. Using a unique dataset, containing ten years of species level length frequency catch data from a multi-gear, multi-species seagrass and coral reef fishery in Kenya, we evaluate species specific fishery statuses, compare gear use to gear regulations and estimate the potential needs for further gear restrictions. Despite the high diversity of the fishery, fifteen species represented over 90% of the catch, and only three species represented 60% of the catch. The three most abundant species in the catch, *Lethrinus lentjan* (Lacepède), *Siganus sutor* (Valenciennes) and *Leptoscarus vaigiensis* (Quoy & Gaimard) all showed evidence of growth overfishing. *Lethrinus lentjan,* with an exploitation rate of 0.82, also shows evidence of recruitment overfishing. Current legal but weakly enforced gear restrictions are capable of protecting a significant portion of the catch up to maturity but optimization of yield will require that the current mesh size be increased from 6.3 to 8.8 and 9.2 cm to increase yields of *L. lentjan* and *S. sutor*, respectively. Given the difficulties of enforcing mesh size, we recommend that the economic benefits of these larger mesh sizes be communicated and enforced through co-management. This abstract is also available in Kiswahili (Abstract S1).

## Introduction

The challenges facing fisheries management are exacerbated where multiple stocks are harvested [1]. This is characteristic of many small-scale fisheries in the tropics, where seagrass beds and coral reefs provide vital protein and livelihoods to many of the poorest countries and communities in the world [2], [3]. However, although small-scale fisheries make up the majority of fisheries globally, scientific investment has historically focused on the relatively few large-scale commercial fisheries where global socio-economic dependence on fisheries is lowest [4]. The combination of social and environmental stressors with limited scientific, financial or institutional support makes managing and assessing seagrass and coral reef fisheries particularly challenging. Consequently, management tends to overemphasize short term, cost effective, and easily controlled tools [5]. Similarly, evaluations of seagrass and coral reef fisheries management typically shy away from nuanced and context specific management in favor of simple assessments and restrictions, which can lead to poor adoption, compliance, or changes in management [6], [7].

Seagrass and coral reef fisheries target multiple species, utilizing multiple gear types. They therefore require flexible approaches to suit the diversity of contexts [8], [9]. Nevertheless, management tends to focus on a limited set of solutions such as capacity reductions, access and gear restrictions. These approaches are rarely popular with fishers, easily adopted by government, and the open-access nature of most fisheries makes these controls difficult to monitor and enforce [6], [10]. Capacity reductions are generally achieved through licensing fishers or vessels or ‘buy backs’ of gear or effort quotas. However, even in industrialized countries, where multiple livelihood options exist, this is challenging and can be highly politicized. Access restrictions, particularly no-take areas, have become the default fisheries management tool in low-income countries [11]. No-take areas are popular due to the relative ease and perceived lower costs of monitoring a small area near shore [12], [13]. Because both capacity reductions and access restrictions involve limiting the number of individuals, time or place of fishing, they can be seen as unethical where dependency is high and livelihood options are limited, and consequently receive little support.

Gear-based restrictions can be designed to modify or adapt a fishery rather than reducing it [14]. This adaptive quality enables gear restrictions to be tailored to the local socio-economic as well as the local ecological context [15]; facilitating a design that for fishers is more acceptable and less intrusive. Gear restriction portfolios can vary considerably and achieve different fishery benefits. Gear restrictions can protect certain species, habitats and reduce the overlap in selectivity across the whole fishery [16], [17], [18]. Management can impose specific requirements on the gear. For example, a minimum mesh size reduces mortality of fish below a certain size, excluder devices reduce bycatch and escape gaps allow certain species or sizes of fish to escape [19], [20]. The diversity of gear management portfolios can allow adjustment to address specific fishery and social-ecological requirements.

Traditional approaches to investigating fisheries management effects on individual species, for example age cohort analysis, require collecting considerable amounts of data particularly in diverse multispecies fisheries such as found on seagrass beds and coral reefs [21]. The available financial and human capital is often limited in many small-scale seagrass and coral reef fisheries. Consequently, the majority of studies investigating multi-species, multi-gear fisheries management effectiveness have tended to focus on community rather than species-level assessments. Although studies have shown generally positive effects of management restrictions at the community level [22], [23], they may mask species and size-specific declines that could undermine reef and fisheries ecology [24]. In addition, the full potential for gear-based management cannot be adequately explored or tested when only evaluated at the community level. Using a unique dataset where species specific length frequency catch composition data were available for a mixed habitat, seagrass and coral reef fishery, we aimed to: 1) examine the status of the fishery by estimating exploitation rates, fishing mortalities, contributions to the catch of individuals below lengths at first maturity (L_mat_) and individuals below lengths to achieve optimum yield (L_opt_); 2) estimate mesh size in use and gear composition to compare with the legal gear restrictions; and 3) estimate the potential that further gear restrictions would have in improving fisheries catches by allowing populations of key fishery species to reach L_mat_ and L_opt_.

## Methods

### Study Site

Small scale artisanal coral reef fisheries of the Indo-Pacific typically exploit mixed habitats including seagrass beds and coral reefs [25]. Fishing in Kenya is representative of these fisheries, conducted from the beach to the fringing reef within the sand, coral and seagrass habitats of the fringing reef lagoon. Fishing pressure is high and from 1997–2007 remained relatively stable, although spatial differences exist [22]. Five main gear types are in operation; beach seine, speargun, trap, net and hand line. Current fisheries laws prohibit the use of beach seine, speargun and any gear with mesh smaller than 6.35 cm (Kenya Gazette Notice No 7565). However, beach seine and spearguns are both in use along the majority of the coastline [22]. Beach seine is a 150–250 meter long net with a mesh of 2–3 cm, actively pulled through the water column by teams of 20–30 men. The selectivity of beach seine creates an overlap in the species caught with other gears, lands substantial numbers of fish <5 cm, damages seagrass beds and corals and repeated use is expected to prevent the resettlement of corals [16], [17], [22]. Although spearguns have the potential to target all species and sizes, this potential is generally not exploited. Spearguns consequently have the least overlap with other gears in species selectivity and land the largest individuals (17–18 cm); they do, however, cause mechanical damage to the habitat [16]. Nets also land large individuals (17–18 cm), while hand lines land the highest trophic level species [16], [23].

### Sampling and Taxonomic Classification

**Figure 1:**
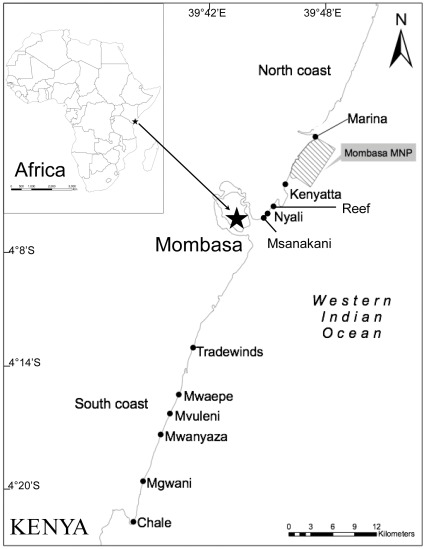
Map of Kenyan coastline. Map of the southern Kenyan coastline showing with locations of the eleven fishery landing sites used in this study.

We sampled eleven sites along a ∼75 km stretch of the southern Kenyan coast line (Fig. 1). We selected sites where predominantly subsistence fishers used a mix of the five main gear types common to the region to land a diversity of seagrass and coral reef species. Four data collectors were used throughout the study, all overlapping for at least one year, enabling training and to ensure consistency in sampling and species identification. At each landing site standard sampling methods, ensuring representation, were used to record gear use, standard lengths, and species [26], [27] of the entire landed catch. Species identification, naming, cross checking and data cleaning were conducted as in [23]. A total of 152 species of fish, recording 27,095 individuals, were sampled in 134 fishing days (ranging from 3–38 for each site), across six sampling years over a 10-year time period (1998–2008) (for site details including sampling days see [23]).

### Catch Composition

Based on cumulative frequency distributions, the 15 most abundant species were found to make up 90% of the catch; these 15 species were therefore retained for further analysis. In order to determine the weight of landed catch for the 15 most abundant species, fish sizes were converted to biomass using standard length-weight relationships from FishBase [28]. We established the proportion of individuals, from the 15 most abundant species, yet to achieve maturity by comparing landed lengths with L_mat_ taken from regional studies where they existed [17]. Where local estimates did not exist, estimates were taken from FishBase [28]. We established the contribution to the catch of each species by each gear type as well as the proportion yet to achieve maturity.

### Life-history Analysis

We determined gear use and its impact on the fishery by calculating each gear’s mean annual (*k*) life-history characteristic (*LH*) (Equation 1) for eight characteristics: intrinsic growth rate (*K*): life span; generation time; natural mortality (*M*); age at maturity; maximum length (*L_max_*); *L_opt_*; and *L_mat_*, of the 15 most abundant species in the catch (*m*). *Y_ik_* is the relative abundance in the catch of species *I,* of *m* species, in year *k*.
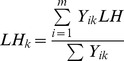
(1)


We analyzed changes over time in the life-history characteristics of each gear type using a Redundancy Analysis (RDA) in CANOCO v4.55. Specifically, we examined the relationship between life-history characteristics and 1) variation in gear groupings through time and 2) species composition. Life-history characteristics were assigned as predictors and species abundance as the response variables [29], [30], [31].

Approaches for quantitatively assessing the state of a fishery are generally data intensive. Therefore, only species that had been sampled in all years, had at least 5000 individuals sampled, and for which sufficient data on the Von Bertalanffy Growth Function (VBGF) coefficients could be found from FishBase were selected for further analysis; this reflected three of the most abundant species in the catch (*Lethrinus lentjan* (Lacepède), *Siganus sutor* (Valenciennes), and *Leptoscarus vaigiensis* (Quoy & Gaimard), which accounted for over 60% of individuals in the total catch.

### Length Frequency Distributions

Recruitment overfishing occurs when there is a reduction of the spawning stock biomass to where recruitment rates are reduced [21]. Growth overfishing occurs at a lower level of fishing to recruitment overfishing and involves removing individuals before they grow to an optimal size [21]. We compared the length frequency distributions of three dominant species in the catch to their L_mat_ and lengths at maximum yield per recruit L_opt_, which allowed us to gauge the percentage of optimal size and mature individuals in the catch and thus provide an indication of whether recruitment and growth overfishing, respectively, were occurring.

### Mortality and Exploitation Estimates

There are several approaches for estimating mortality and they all make assumptions that may not be reasonable [32]. We examined the status of the fishery by calculating rates of instantaneous total mortality (Z) for the top three species in the catch (*L. lentjan, S. sutor,* and *L. vaigiensis*) using the linearized length converted catch curve method as described by Pauly [33]. When the available data is limited, but mean length above first capture (L_c_) and an independent maximum possible length (L_∞_) exist, Z can be calculated using a modified method of Beverton & Holt [34] that is available in FishBase. We therefore compare the results obtained by these two methods.

### Linearized Length Converted Catch Curve Method

We calculated mortality rates based on the age of individuals in the landed catch. The basic theory behind age-based mortality estimates is that as a cohort ages, the decrease in the number of survivors reflects the number of individuals dying from natural and fishing mortality; Z. However, we first needed to establish the age of individuals in the catch by converting length-frequency data (*L_t_*) to age-frequency data (*t*) using the inverse of the VGBF [33] (equation 2). Sizes close to the asymptotic limit increase the error associated with length-age conversions; they were therefore excluded. We used life history characteristics - *L_∞_*, *K*, and time zero (*t_0_*) - from regional studies where they existed, because these are a better reflection of local characteristics, they generate more accurate age estimates.
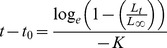
(2)


Where regional estimates did not exist, *L_max_* of the sampled catch was used to calculate *L_∞_* [33] (equation 3).
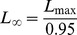
(3)


The decrease in the number of survivors, or *Z*, can be established from the exponential rate of decline of individuals in the catch. We therefore plotted the natural logarithm of the number of fish in the catch (*N*) against their age (*t*) (equation 4). We used this curve to calculate (*Z*) from the descending slope of the best fit line (*b*), using least squares linear regression. However, first the number of fish in the catch at a particular age needed to be adjusted to account for differences in the length of time spent in a size class as fish grew (*t*) (equation 5) [33]. Because this approach assumes Z is constant beyond some reference age, we can only use individuals that have fully recruited to the fishery. The age that individuals have fully recruited to the fishery is represented by the point when the catch curve begins to decline at a steady rate; we therefore base the regression line on individuals beyond this point.
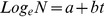
(4)

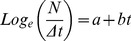
(5)


There are some implicit assumptions in this approach that require caution. The main danger is if recruitment is declining, Z will be under estimated.

### Modified Beverton & Holt [33] Method in Fishbase

We recalculated *Z* in Fishbase (Equation 6), using region specific *K*, average length of the landed catch (*L_mean_*) and lengths at first capture (*L_50%_*) (see next section).
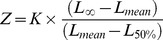
(6)


This approach assumes steady state conditions and infinite exploitable life spans. However, with short lived species, or when the fishery life span is less than theoretical, Z will be overestimated [35].

We calculated two exploitation rates (*E*) for each species, one based on values of *Z* obtained from the Beverton & Holt method in Fishbase and the second based on values of *Z* obtained from the method in Pauly [33]. For both methods we first calculated fishing mortality (*F*) (Equation 7), then *E* (equation 8). We used estimates of *M* from recalculations in FishBase based on region specific measures of *L_∞_* and *K* where they existed.
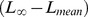
(7)

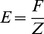
(8)


We established the status of the stock by comparing the calculated values of E with optimum exploitation rates (E_opt_) based on the assumption that E_opt_ is about equal to 0.5. The use of 0.5 for E_opt_ assumes that the sustainable yield is optimized when F = M [36]. Therefore, when E is greater than E_opt_ the stock is overfished, and when E is less than E_opt_ the fishery is under exploited.

### Selectivity and Mesh Sizes

In order to establish potential sources of overfishing, we calculated mesh size in use for each of the three most abundant species and compared these estimates to the legal minimum mesh size. We were also interested in what further restrictions would be needed to allow key fishery species to reach L_mat_ and L_opt_. We therefore also calculated mesh size needed to protect the top three species in the catch up to L_mat_ and L_opt_.

Mesh size estimates are calculated using species selectivity and lengths at first capture (L_50%_). We calculated L_50%_ using the left-hand side of the linearized catch curve, which accounts for differences in time spent in each age class as a fish grows, to derive gear selection ogives following [37]. A gear selection ogive is the logistic curve representing the fraction of catch retained, plotted against the mid length of the corresponding length group (i.e. cumulative frequency distribution). This assumes that the change in the number of fish retained by a certain mesh size as fish size increases is described by the logistic curve (equation 9), where *P* is the proportion of the total catch of length *L* caught, *r* is a constant and *L_50%_* is the mean length at which 50% of the fish are retained [37], [32]:
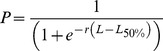
(9)


We established independent selection factors (*SF*) for the top three species in the catch, using the nomogram in [33], which is based on species specific depth ratios (standard length/total length) obtained from FishBase. Mesh size in use (*MS_use_*), mesh size needed to protect species below length maturation (*MS_mat_*), and to lengths to achieve optimum yield (*MS_opt_*), were then calculated using a back calculation of the selection factors calculation (equation 10), based on our independently established SF and either *L_50%_* (equation 11), *L_mat_*, (equation 12) or *L_opt_* (equation 13) [37].
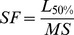
(10)

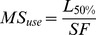
(11)

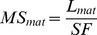
(12)

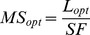
(13)


### Ethics

Research clearance, which includes any relevant ethics clearance, was provided by Kenya’s National Council for Science and Technology under research permit number NCST/RRI/12/1/BS/209.

## Results

### Catch Composition

The three most commonly landed species were *Lethrinus lentjan, Siganus sutor* and *Leptoscarus vaigiensis.* These three species combined made up 63% by abundance and 75% by weight of the 15 most abundant species (Table 1). *L. lentjan* was the species most often caught (22% of landed fish), although *S. sutor* contributed considerably more to the catch by weight (45% of landed weight) (Table 1). Forty seven percent of total landed catch were immature individuals of these three species (Table 1).

**Table 1:** Catch composition and proportion under lengths at first maturity. Percentage of total catch by weight and number for the 15 most commonly caught species, and percentage under length at first maturity (L_mat_).

Species	Weight(%)	Number(%)	Number<L_mat_(%)
***Lethrinus lentjan***	**13.2**	**22.0**	**21.7**
***Siganus sutor***	**45.1**	**21.7**	**17.4**
***Leptoscarus vaigiensis***	**16.6**	**18.6**	**8.1**
*Calotomus carolinus*	4.4	5.1	4.1
*Lethrinus harak*	5.9	4.2	3.4
*Lutjanus fulviflamma*	2.5	2.9	1.6
*Acanthurus triostegus*	1.9	2.6	0.5
*Parupeneus macronemus*	1.7	2.2	1.2
*Cheilio inermis*	1.2	2.1	1.8
*Scarus psittacus*	1.8	1.6	0.6
*Parupeneus barbarinus*	0.9	1.6	1.0
*Lethrinus obsoletus*	0.9	1.5	1.4
*Acanthurus nigrofuscus*	1.2	1.4	1.2
*Cheilinus trilobatus*	1.9	1.3	1.1
*Gerres oyena*	0.8	1.1	0.9

Eighty percent of the total catch and 81% of the top three species were caught by either beach seine, net or trap; all fishing gears that employ a mesh, leaving only 20% of the catch not influenced by mesh size (Table 2). Beach seine nets landed the greatest number of fish (over 40% of the catch), followed by traps (19.9%), nets (11.5%), hand line (9.4%) and spear (8.7%). Beach seine landed the greatest proportion of individuals that were under L_mat_ (79.3%), followed by hand line (67.2%), with nets landing the smallest proportion of individuals under L_mat_ (51.4%).

**Table 2:** Catch composition, and proportion under lengths at first maturity by gear type.

Species	Catch					<L_mat_				
	Beach seine	Trap	Net	Line	Spear	Beach seine	Trap	Net	Line	Spear
***Lethrinus lentjan***	**9.8**	**4.9**	**1.4**	**5.7**	**0.3**	**99.6**	**99.4**	**93.3**	**98.7**	**90.7**
***Siganus sutor***	**8.2**	**8.9**	**3.6**	**0.1**	**0.9**	**93.9**	**75.2**	**56.3**	**86.2**	**94.6**
***Leptoscarus vaigiensis***	**10.3**	**2.6**	**1.0**	**0.1**	**4.6**	**54.3**	**25.3**	**49.3**	**36.0**	**28.9**
*Calotomus carolinus*	2.9	0.9	0.6	0.0*	0.7	97.1	61.5	65.3	38.5	59.8
*Lethrinus harak*	1.9	0.2	1.5	0.4	0.2	80.9	95.2	72.7	93.9	80.3
*Lutjanus fulviflamma*	0.8	0.4	0.5	0.8	0.4	80.0	28.6	27.8	71.4	41.3
*Acanthurus triostegus*	0.7	0.6	1.1	0.1	0.2	28.9	25.5	13.0	4.5	21.4
*Parupeneus macronemus*	1.1	0.3	0.5	0.2	0.2	68.7	23.8	36.7	47.1	44.4
*Cheilio inermis*	0.9	0.1	0.1	0.8	0.3	91.1	85.0	71.9	82.4	65.2
*Scarus psittacus*	1.0	0.2	0.2	0.0*	0.2	47.6	13.2	11.8	ND*	14.0
*Parupeneus barbarinus*	0.6	0.6	0.3	0.0*	0.1	89.1	34.2	64.1	100.0	71.4
*Lethrinus obsoletus*	0.5	0.1	0.1	0.8	0.0*	93.0	90.3	65.4	100.0	62.5
*Acanthurus nigrofuscus*	0.2	0.2	0.4	0.0*	0.5	98.4	87.0	82.3	54.5	85.2
*Cheilinus trilobatus*	0.4	0.2	0.1	0.4	0.2	79.0	88.9	61.8	93.8	74.6
*Gerres oyena*	1.0	0.0*	0.1	0.0*	0.0*	87.9	ND*	0.0*	33.3	0.0*
***Total***	40.3	19.9	11.5	9.4	8.7	79.3	59.5	51.4	67.2	55.6

Contribution (%) to total catch of the top 15 species by each major gear type, and proportion (%) of fish landed by each gear type that are under lengths at first maturity.

* ND - no data in catch; 0.0 is not necessarily absolute zero.

Across all gears, over 90% of the landed *L. lentjan* were below L_mat_, with 99.6% of those landed by beach seine under L_mat_ (Table 2). Over 50% of landed *S. sutor* were below L_mat_ across all gears and over 90% of individuals landed by beach seine and spears were below L_mat_ (Table 2). A considerably smaller proportion of individuals of *L. vaigiensis* were caught below L_mat_, with 25% across all gears and a maximum of 54.3% for beach seine (Table 2). Of the remaining 12 species comprising the top 15 species in the catch, beach seine landed a high proportion of several species below L_mat_; including over 90% of *Acanthurus nigrofuscus*, *Calotomus carolinus*, *Lethrinus obsoletus* and *Cheilio inermis* individuals under L_mat_ (Table 2). Also of note, 100% of landed *Parupeneus barberinus* and *L. obsoletus* were caught below L_mat_ by line fishing (Table 2). It must be noted however, that some of these species caught by specific gears make up a very small proportion of the catch (Table 2).

### Life History Characteristics

**Figure 2:**
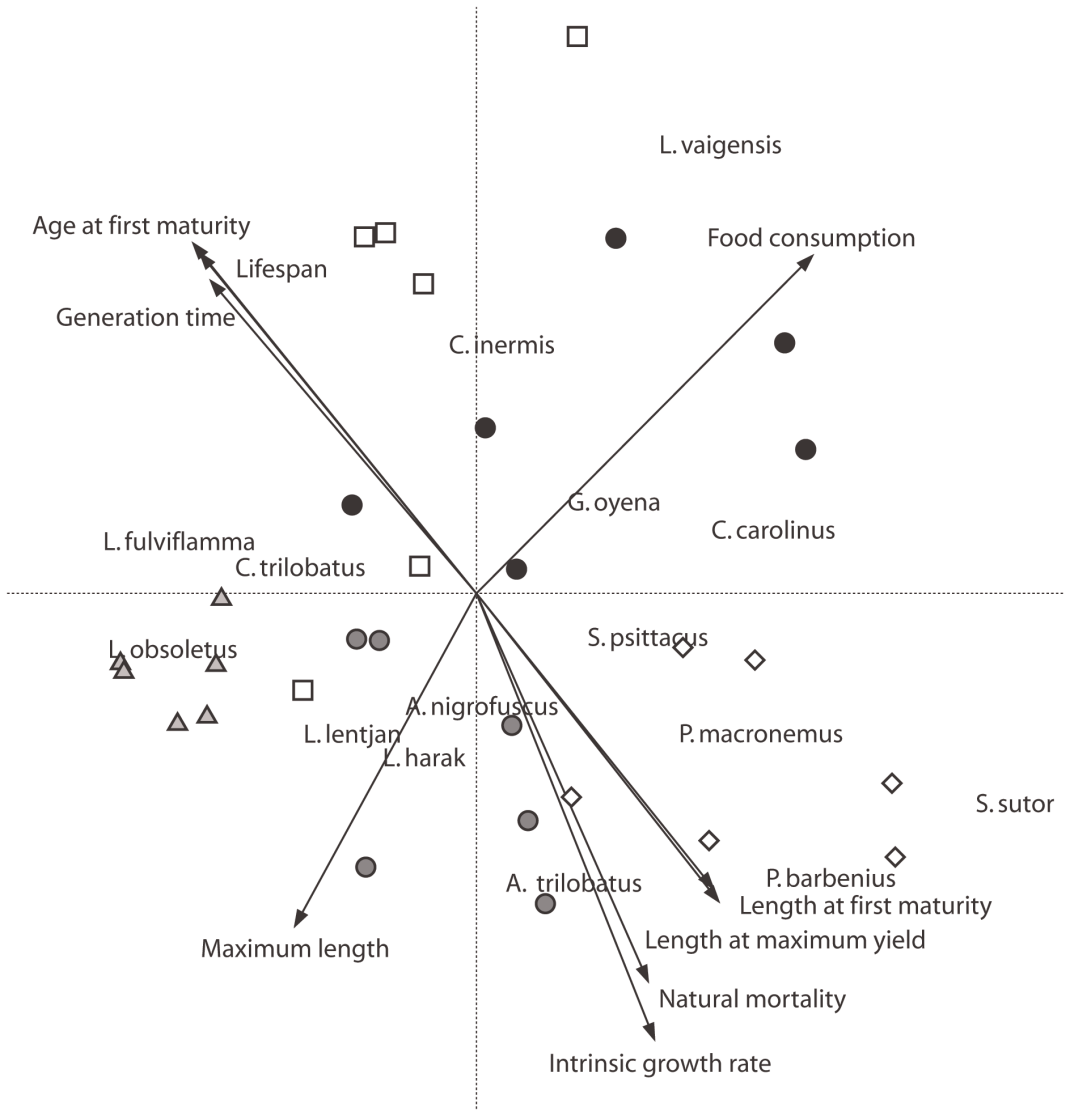
Distribution of species and life history parameters by gear. A redundancy analysis showing species distribution by gear, over ten years, along the Kenyan coast; with the associated life history characteristics driving differences in gear. Replication is at the level of a year; p = 0.002, F = 6.60, C1 = 49.1%, C2 = 32.4%. Black circles- Beachseine; white square- Spearguns; grey triangle- Hook and line; white diamond- Trap; grey circle- Net; arrows - life history parameters.

Redundancy analysis suggests individual gears have differential influences in selecting for species and their associated life-history characteristics (Fig. 2). The compositions of spear and beach seine catches clustered loosely together and were distinct from hand line, trap and net catches (Fig. 2). The beach seine catch was composed of herbivores with greater food consumption while spearguns caught species with longer life spans, age at maturity and generation time. This is contrasted with a lower consumption rate of species caught by line, trap, and net fisheries where L_mat_, L_opt_, L_max_, M, and K were all greater.

### Length Frequency Distributions

**Figure 3:**
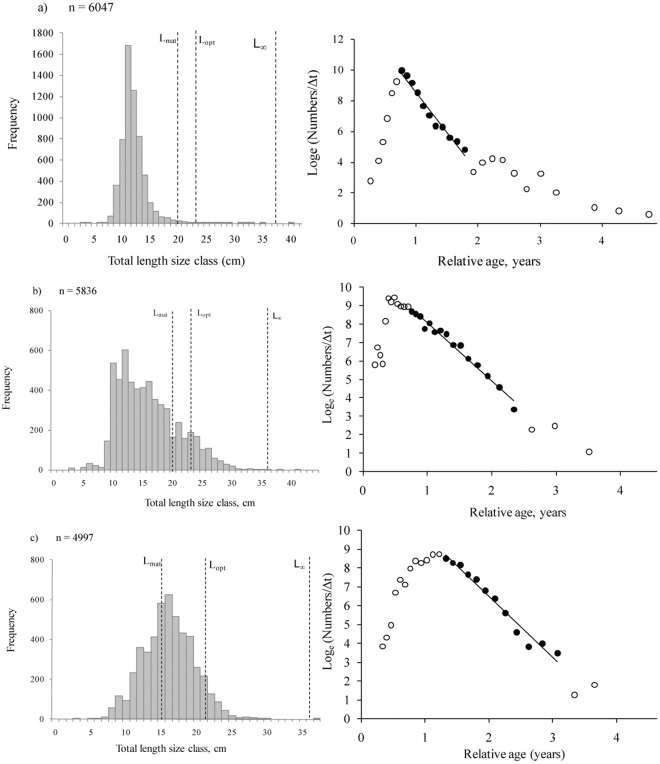
Size frequency distribution and mortality curves. Size frequency distribution with length at maturity (L_mat_), length to achieve optimum yield (L_opt_), and length at infinity (L_∞_) overlain; and mortality curves calculating Z for; a) *L. lentjan (*y = 13.83−5.26x, r2 = 0.97); b) *S. sutor (*y = 11.22–3.15x, r2 = 0.98) and; c) *L. vaigiensis (*y = 12.98–3.24x, r2 = 0.97).

Overall, one percent of the landed *L. lentjan* were above both L_mat_ and L_opt_ (Fig. 3a), suggesting both growth and recruitment overfishing of *L lentjan* is occurring. Thirteen percent of the landed *S. sutor* were above L_opt_ and 20% above L_mat_ (Fig. 3b), again suggesting that both growth and recruitment overfishing of *S. sutor* is occurring. Four percent of the landed *L. vaigensis* were above L_opt_ and 56% above L_mat_ (Fig. 3c) suggesting growth overfishing, but not recruitment overfishing, is occurring for this species.

### Mortality and Exploitation

The life history characteristics used in the mortality estimates based on the catch curve method obtained Z estimates of 5.26, 3.15, and 3.24 for *L. lentjan, S. sutor* and *L. vaigiensis* respectively (Fig. 3, Tables 3,4). These Z estimates equate to F estimates of 4.29, 1.66, and 2.26 (Table 4). F estimates can be understood with the help of conversion tables that convert F values to their equivalent % removed in a year. F estimates of 4.29, 1.66, and 2.26 mean that of all the *L. lentjan, S. sutor* and *L. vaigiensis* individuals present in the lagoon in a year, 98%, 81%, and 89% respectively were removed by fishing (Table 4).

**Table 3:** Life history characteristics used.

Species	L_∞_ (cm)	K	t_0_ (yrs)	M	L_mean_(cm)	L_mat_ (cm)	<L_mat_ (%)	L_opt_ (cm)
*L. lentjan*	37.3^*^	0.48	-0.30	0.97****	12	20.3***	99	23.1****
*S. sutor*	36.2**	0.87**	-0.24	1.49****	16	20.2***	80	23.0****
*L. vaigiensis*	36.6	0.49	-0.32	0.98****	16	15.1***	44	22.7

Life history characteristics used in mortality estimates for the three most abundant species in the catch. Parameter estimates taken from Fishbase except where indicated. L_∞_ = Maximum possible length. K = intrinsic growth rate. t_0_ = hypothetical time zero. M = mortality. L_mean_ = mean length L_mat_ = length at first maturity. L_opt_ = length at maximum yield per recruit.

** Ntiba (1989).

*** Mangi & Roberts (2004).

**** recalculated using new L_∞_ in fishbase.

**Table 4:** Mortality and exploitation estimates.

Species	Pauly (1983)			Beverton & Holt (1956)				Status
	Z_catch curve_	F_catch curve_	E_catch curve_	Z_Fishbase_	F_Fishbase_	E_Fishbase_	E_opt_	
*L. lentjan*	5.26	4.29	0.82	11.04	10.07	0.91	0.5	over exploited
*S. sutor*	3.15	1.66	0.53	3.74	2.25	0.60	0.5	over exploited
*L. vaigiensis*	3.24	2.26	0.70	4.21	3.23	0.77	0.5	over exploited

Mortality and exploitation estimates based on the linearized length converted catch curve method [23] and the modified Beverton and Holt method as in FishBase for the three most abundant species in the catch.

The mortality (F & Z) and exploitation (E) estimates obtained using the Beverton & Holt method in FishBase all exceeded the estimates derived from the catch curve method, and for *L. lentjan* more than doubled these estimates (e.g. F = 5.26 compared to F = 11.04) (Table 4). The discrepancy between the two methods is likely due to the assumption of infinite exploitable lifespans in the Beverton & Holt method and the reality of a short fishery life span in a highly exploited fishery. Although the FishBase approach does contain a modification to the Beverton & Holt method, mortality estimates obtained from FishBase should still be used with caution where a fishery is known to be short lived and heavily exploited [35]. The E rates obtained by both methods were above optimal (i.e. >0.5) for all species; E for *S. sutor* was the closest to optimum at 0.53 (Table 4).

### Selectivity and Mesh Sizes

**Figure 4:**
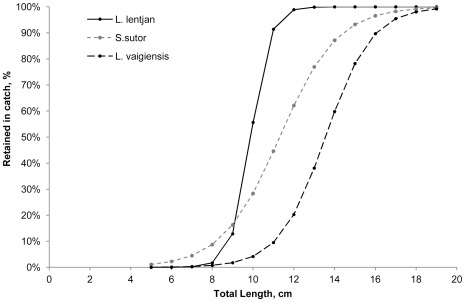
Species selection ogives. Logistic curve selection ogives for the three most commonly landed species in the catch, showing percent retained in the catch by body length.

The catch of the three most abundant species landed indicates that the mesh size used in the fishery is ∼5 cm, compared to a legal minimum mesh size of 6.35 cm (Table 5). L_50%_ was 10.9 cm, 11.3 cm, and 13.6 cm for *L. lentjan, S. sutor* and *L. vaigiensis* respectively (Fig. 4, Table 5). These were based on SF of 2.3, 2.2 and 2.4 derived from nomograms. If managers wanted to increase protection of the three most abundant species in the catch to ensure L_50%_ was above L_mat_, mesh regulations would have to be increased to 8.8 cm and 9.2 cm for *L. lentjan* and *S. sutor* respectively and enforced at 6.3 cm for *L. vaigiensis* (Table 5). In order to increase protection of the three most abundant species in the catch to ensure L_50%_were above L_opt_, mesh regulations would have to be increased to 10.0 cm and 10.5 cm and 9.2 cm for *L. lentjan, S. sutor, and L. vaigiensis* respectively (Table 5).

**Table 5:** Management metrics for mesh in use and potential further restrictions.

Species	L_50%_	SF	Mesh_used_	Mesh_Lmat_	Mesh_Lopt_
*L. lentjan*	10.9	2.3	4.74	8.8	10.0
*S. sutor*	11.3	2.2	5.14	9.2	10.5
*L. vaigiensis*	13.6	2.4	5.65	6.3	9.2

Calculated lengths at which 50% of each species are retained in the catch, selectivity factors, mesh size used, required mesh legislation to protect species up to L_opt_ and L_mat_ for the three most abundant species in the catch.

## Discussion

Although gear-based management has been shown to increase catch per unit effort of seagrass and coral reef fisheries in Kenya [22], [23], the top three target species were fully exploited. We found the most abundant species in the catch, *Lethrinus lentjan*, to be grossly over exploited and it is likely that less commonly landed sibling species, such as *Lethrinus obsoletes* (Forsskål), experience similarly high exploitation levels. The second and third most abundant species, representing nearly half of the catch, *Leptoscarus vaigiensis* and *Siganus sutor*, are also over exploited. Our estimates for mesh sizes in use were all below the legal limit and prohibited gears are still in use, suggesting the need for better enforcement. However, the scientific basis for restricting spearguns and beach seines in Kenya is sound and we put forward two justifications for this. Firstly, multi species fisheries often exploit different species and life history traits at different rates [38], [39]; we found prohibited beach seine nets and spearguns to target slower growing species that are likely to be less resilient to the high fishing pressures seen in Kenya. Secondly, the legal minimum mesh size (6.35 cm) would allow *L. vaigiensis* individuals to mature. Enforcing the current gear restrictions would therefore protect slow growing species and juvenile *L. vaigensis*. The legal minimum mesh size would need to be increased by 2.45 cm and 2.85 cm respectively to protect immature *L. lentjan* and *S. sutor* individuals from fishing. Increases this large may need to occur gradually to avoid a period of lost catch. Therefore alternate management measures, such as 6-km^2^ closures which benefit *L. lentjan* and *S. sutor* species in adjacent fisheries [40], will be needed to provide insurance to juveniles of these taxa.

There is a need for increased enforcement efforts as Kenya’s current gear-based restrictions are justified based on life history and body sizes. Gears in this fishery have been previously shown to target specific species and sizes that reduce the overlap in species selectivity, thereby minimizing the potential to overexploit species or sizes through competition between gears [16]. Here, we find further evidence that gears also selectively target specific life history characteristics, suggesting management could be targeted to alleviate pressure on the life history characteristics that are most susceptible to overfishing [41]. Effective management should ideally aim to conserve species that are slow growing, late maturing, have lower rates of mortality and larger lengths at maturity [41], [42], [43], [44]. Beach seines and spears, currently restricted though commonly used, target species characterized by a slower turnover. In addition to gear bans, size restrictions present a relatively simple form of management that can be monitored from the shore. Eighty percent of the beach-seine catch and 60% of the total catch, is composed of individuals yet to achieve maturity, suggesting there is a need for length-based management. Furthermore, 80% of the total catch and 81% of the catch of the three most commonly landed species were caught with beach seines, nets, or traps; all gears where catch can be controlled through mesh size restrictions, presenting a simple and powerful tool to address overfishing.

Although holistic approaches to management, incorporating inter-specific interactions, are needed for sustainable fisheries [4], these approaches do not preclude the need for single species assessments and targets. Indeed, it is common for tropical multi-species fisheries to be dominated by a few key species [45], [46]. Kenya’s seagrass and coral reef fisheries are dominated by only three species, which represents 63% of the catch. The same three species were among five species that comprise >60% of the catch in Mozambique [46]. Although a multi-species fishery may show signs of overall recovery or stability, individual stocks key to the fishery as a whole may still be in decline. For example, recent studies show Kenya’s fishery recovering post 2002, yet we find the most commonly landed species in the catch, *L. lentjan,* to be over exploited and only responding positively near fisheries closures [40]. Ranked third in its contribution to the catch by weight, *L. lentjan* has the greatest mortality and exploitation estimates (F = 4.29, E = 0.82). Nearly all of the landed *L. lentjan* individuals are yet to reach optimal or lengths at maturity, suggesting both “growth overfishing” (taking too many fish when they were too small, biologically and economically; i.e. below L_opt_) and “recruitment overfishing” (taking too many fish when they are reproductively immature, so that recruitment is impaired [21]) is occurring. These mortality and exploitation figures are the highest reported in the literature. Studies from the region assessing trap fisheries landings of *Lethrinus mahsena* (Forsskål), a species with similar life history characteristics, report comparable but lower mortality and exploitation (F = 2.48, E = 0.64) [47]. Studies that estimate *L. lentjan* mortality and exploitation rates, from regions with far lower fishing pressures and larger fishing areas, report considerably lower values (Arabia Z = 0.44, F = 0.22, Great Barrier Reef Z = 0.18, Seychelles Z = 0.142) [38], [48], [49].

With such high exploitation rates, the *L. lentjan* fishery in Kenya should have collapsed, and although *Lethrinid* catches have declined [47], they were still a significant proportion in the catch, with the average landed lengths of individuals having increased with management restrictions [23]. Two explanations are possible; 1) our study may have failed to adequately sample the available population, 2) our study may have sampled the available population, but this population is receiving replenishment from outside the fished area. Although the first hypothesis is plausible, the gears used in the fishery are capable of catching individuals larger than the asymptotic length of *L. lentjan.* Furthermore, our size frequency distribution contains individuals up to the asymptotic length of *L. lentjan*, suggesting our sampling was adequate. It is more likely that there is an ontogenetic migration of *L. lentjan* individuals from seagrass beds to deep reefs [50], [51], with juveniles and small adults found principally in seagrass beds, mangroves and sandy areas whilst adults are solitary and found in deeper water [23], [28], [50]. This cross ecosystem separation of the adult and juvenile stages has been suggested as a strategy to minimize mortality and maximize growth [52], [53]. The presence of mangrove and seagrass habitats in proximity to deeper reefs have been identified as important factors in affecting reef assemblages [54] and enabling resettlement of the pelagic larval stage onto suitable habitats [55]. As fishing in Kenya occurs predominantly in the shallow coral, sand and seagrass lagoon, there is possibly a natural refuge from fishing for the adult *L. lentjan* stock in deeper reef habitats. The natural protection provided to these deeper habitats may therefore be critical for replenishing the lagoon fishery [55], [56]. Interestingly, lethrinds and siganids were one of the main beneficiaries of fisheries closures in this region, which indicates the importance of refuge, and that closures are another way to protect these valuable taxa [40], [56].

*Siganus sutor* contributes the most to the catch by weight and is the second most commonly landed species in the catch. Fishing and exploitation rates for *S. sutor* were the lowest of the three species, however a large proportion of the *S. sutor* catch was yet to mature suggesting that growth overfishing is likely to have occurred. Although the status of the stock is over exploited, it is conceivable that this fishery could be brought within optimally exploited status if mesh size and gear exclusion laws are enforced, particularly because this species is very fast-growing. Our exploitation and mortality rates compare well with earlier studies in Kenya (Z = 2.59, F = 1.44, E = 0.56) [47], but remains below some estimates from the region (e.g. Seychelles Z = 8.6) [38].

*Leptoscarus vaigiensis* is the third most commonly landed species in the catch by number and weight. Although a greater proportion of landed *L. vaigiensis* reached maturity than *L. lentjan*, *S. sutor* or most of the top 15 species, fishing mortality and exploitation estimates remain greater than *S. sutor* and the stock was over exploited. However, there is a dearth of comparable data on *L. vaigiensis* exploitation, and no region-specific life history parameters. Regional values are critical for assessing fishery status as fishing can and does alter life history parameters. When high fishing pressures reduce population size, there is an increase in the relative abundance of food, resulting over time, in faster growth, smaller asymptotic size, and smaller size at first maturity [57]. For example, fishing pressures are related to decreases in length at maturity in a number of fisheries [42], [58]. There is a need, therefore, for regional studies to assess key life history parameters particularly for *L. vaigiensis*, a key species in Kenyan seagrass and coral reef fisheries [46]. Relatively small changes in the VGBF parameters can alter the results and subsequent interpretations. Therefore, it is likely that with better, region specific parameters, mortality estimates would change. These and other uncertainties inherent in fishery assessments reinforce the need to combine approaches when assessing the status of a fishery [21].

### Management Recommendations

Beach seine and spears were still commonly used, with almost half of the fish landings attributable to beach seine catches. Furthermore, estimates of the mesh size in use in the fishery are more than one centimeter below legal requirements. Yet, there is still the opportunity for Kenya’s fisheries management to build on recent fishery successes [22] by focusing on: 1) improving compliance with current gear restrictions; and 2) implementing complimentary approaches that provide refuge to specific species and life history stages. Compliance may be improved through mechanisms such as increasing monitoring that ensures penalties are appropriate and facilitating channels to resolve conflicts when they arise [59]. In addition, locally relevant education and awareness that illustrates the economic benefits of management could also improve compliance. For example, beach seine exclusions have proven successful in certain locations along the Kenyan coast [40]. Promoting the lessons learned from these localized successes in areas where compliance is low could catalyze changes elsewhere. However, because the current top-down management is not working effectively, there is a need for government agencies to promote greater education and co-management arrangement that foster local responsibility [58]. Fostering these relationships encourages the adaptive and flexible approach required to balance multiple objectives in an environment that, by necessity, will continue to be heavily modified by human use but could produce more income for impoverished fishers if managed more effectively.

Enforcing the current mesh limits would allow immature *L. vaigensis* individuals to mature. This would in turn increase the reproductive potential of *L. vaigensis*, thus increasing yield over time. In order to provide immature *S. sutor* or *L. lentjan* individuals similar levels of protection a minimum mesh size of 8.8 cm and 9.2 cm would be necessary (an increase of 2.45 cm and 2.85 cm from the current legal limit, and over 3 cm from that in use); a challenging target in the local socio-economic context. Consequently, current approaches to management are unlikely to maintain the resilience of the whole system or optimize incomes [22], [59], [40]. Alternate, complementary approaches are therefore necessary to maintain key life stages including juvenile *S. sutor* or *L. lentjan*; key habitats such as seagrass or mangrove nursery grounds; functional groups [60]; trophic levels [61]; and species not protected within current gear restrictions [18]. The current system of marine parks along the Kenyan coastline goes some way to achieving these goals [59]. The role deeper unexploited areas play in the potential ontogenic migration of *L. lentjan* could provide further strategic areas for protection.

### Conclusion

Kenya’s current legal gear restrictions have the potential, if enforced, to increase fishery yields [22] and protect slow life history traits [23]. However, they are insufficient at protecting against species and functional losses; characteristics necessary for of resilient systems [24]. Modifications to current restrictions [62] and adaptive approaches to gear management [18] will move the fishery in a positive direction away from the current social-ecological poverty trap. The proposed changes are likely to be received more favorably if they are integrated into education and emergent local institutional structures [63] and the scientific basis for management decisions or modification is included in local decision-making processes. However, these approaches will still need to be complemented by a system of refugia, established to protect key habitats, life histories and species identified as exploited by the fishery, but not covered by gear restrictions [40]. In this way, fisheries management may take a ‘toolbox’ approach [5], tailoring a diversity of tools to a portfolio of needs.

## Supporting Information

Abstract S1 Kiswahili abstract.
(DOCX)
