# Supplementary material for: Assessing Gear Modifications Needed to Optimize Yields in a Heavily Exploited, Multi-Species, Seagrass and Coral Reef Fishery
Source: PLoS One. 2012 May 4;7(5):e36022. doi: 10.1371/journal.pone.0036022 (PMC3344850; doi:10.1371/journal.pone.0036022)
Supplement: Abstract S1 — Kiswahili abstract. (DOCX) [file pone.0036022.s001.docx]

**MUHTASARI**

Usimamizi bora wa uvuvi ni muhimu ikiwa wavuvi wadogo, hasa wale wanaovua kwenye miamba ya matumbawe na maeneo ya manyasi baharini, wataendelea kukimu mahitaji ya chakula kwa jamii maskini zaidi ulimwenguni. Usimamizi unaolenga zana za uvuvi haujawakilishwa kikamilifu na kufanyiwa utafiti wa kutosha. Ingawa hivyo, una uwezo wa kuwa mbadilifu ili kushughulikia malengo tofauti na kulenga mahitaji ya mandhari husika ya kijamii na kiuchumi. Usimamizi fanisi wa uvuvi kwenye miamba ya matumbawe na maeneo ya manyasi umetathminiwa kwa kuangalia vile jamii ya samaki ilivyo kwa ujumla. Lakini jamii ya samaki kama kiashiria inaweza kuficha upungufu ulioko kwa aina tofauti ya samaki ambao wanachangia sehemu kubwa ya mazao na mapato ya uvuvi. Tumetumia maelezo ya kipekee, yanayojumulisha miaka kumi yakiangazia vile urefu na wingi wa samaki wa aina tofauti wanaovuliwa kutumia zana mbalimbali za uvuvi kwenye miamba ya matumbawe na maeneo ya manyasi kwenye bahari ya Kenya. Kutumia maelezo haya, tumetathmini hadhi za uvuvi wa aina maalum ya samaki, tukalinganisha utumizi wa zana za uvuvi na kanuni zilizoko na pia tukakisia uwezekano wa mahitaji ya kuendela kuwekea vikwazo aina fulani ya zana za uvuvi. Licha ya kiwango cha juu cha aina tofauti ya samaki, aina 15 waliwakilisha asilimia 90 ya samaki waliopatikana na wavuvi, na aina 3 ya samaki waliwakilisha asilimia 60. Aina tatu ya samaki wanaovuliwa kwa wingi zaidi, changu, tafi na pono, wote walionyesha kuwa wanavuliwa kupita kiasi kulingana na ukuaji wao. Changu anavuliwa kwa kiwango cha 0.82 na kuna ushahidi wa kwamba anavuliwa zaidi kushinda uwezo wake wa kujisajili. Sheria ya sasa, ambayo imetekeleza kidhaifu kuwekea vikwazo zana fulani za uvuvi, inauwezo wa kulinda sehemu kubwa ya samaki hadi wakomae bila kuvuliwa. Lakini kuboresha mavuno ya uvuvi kutahitaji kiwango cha kichungi cha nyavu kiongezwe kutoka 6.3 mpaka 8.8 na 9.2 ili kuongeza mapato ya Changu na Tafi. Kutokana na ugumu wa kuhakisha kichungi cha nyavu kimefuatiliwa, tunapendekeza kwamba kuwe na mawisiliano kuhusu faida ya kiuchumi ya nyavu ambazo vichungi vyake vina nafasi kubwa na utekelezaji wa pendekezo hili upitie kwa njia ya ushirikiano wa usimamizi wa maliasilia.
